# Photo thermal effect graphene detector featuring 105 Gbit s^−1^ NRZ and 120 Gbit s^−1^ PAM4 direct detection

**DOI:** 10.1038/s41467-021-21137-z

**Published:** 2021-02-05

**Authors:** S. Marconi, M. A. Giambra, A. Montanaro, V. Mišeikis, S. Soresi, S. Tirelli, P. Galli, F. Buchali, W. Templ, C. Coletti, V. Sorianello, M. Romagnoli

**Affiliations:** 1 Tecip Institute – Scuola Superiore Sant’Anna, Pisa, Italy; 2 Photonic Networks and Technologies Lab – CNIT, Pisa, Italy; 3 Center for Nanotechnology Innovation @NEST - Istituto Italiano di Tecnologia, Pisa, Italy; 4 Graphene Labs, Istituto Italiano di Tecnologia, Genova, Italy; 5 Fondazione INPHOTEC, Pisa, Italy; 6 Nokia Solutions and Networks Italia, Vimercate, Italy; 7 Nokia Bell Labs, Stuttgart, Germany

**Keywords:** Integrated optics, Optoelectronic devices and components

## Abstract

One of the main challenges of next generation optical communication is to increase the available bandwidth while reducing the size, cost and power consumption of photonic integrated circuits. Graphene has been recently proposed to be integrated with silicon photonics to meet these goals because of its high mobility, fast carrier dynamics and ultra-broadband optical properties. We focus on graphene photodetectors for high speed datacom and telecom applications based on the photo-thermo-electric effect, allowing for direct optical power to voltage conversion, zero dark current, and ultra-fast operation. We report on a chemical vapour deposition graphene photodetector based on the photo-thermoelectric effect, integrated on a silicon waveguide, providing frequency response >65 GHz and optimized to be interfaced to a 50 Ω voltage amplifier for direct voltage amplification. We demonstrate a system test leading to direct detection of 105 Gbit s^−1^ non-return to zero and 120 Gbit s^−1^ 4-level pulse amplitude modulation optical signals.

## Abstract (web summary)

The fast carrier dynamics and ultra-broadband optical properties of graphene make it suitable for optical communications. Here, the authors demonstrate a photo-thermo-electric graphene photodetector integrated on a Si waveguide featuring 105 Gbit s^−1^ non-return to zero and 120 Gbit s^−1^ 4-level pulse amplitude modulation direct detection.

## Introduction

The global number of devices connected to the IP network is growing at a compound annual growth rate (CAGR) of 10%, and is expected to exceed 28 billion connected devices by 2023^1,2^. The speed of these connections will also increase, with 5G devices expected to reach 575 Mbps in wireless links for the broad community of users by 2023^1^. As a result, next optical network and interconnect technologies will be developed to meet the increasing bandwidth connectivity demands. One of the next major transitions will be the general availability of 400-Gigabit Ethernet (GbE) technology^2^, along with the development and standardization of the 800-Gigabit Ethernet (GbE) technology for optical transceivers providing higher capacity at a reduced cost, footprint, and power consumption^3^. Even more demanding the sixth generation of the wireless network (6G) will require >1000 Gb s^−1^ per user and beyond^4^.

In this scenario, Si photonics technology plays an essential role as it offers the possibility of cost-effective large volume production, thanks to the availability of well-established silicon fabrication facilities and the relatively low cost and high abundance of the material^5,6^. Si photonics transceivers have reached the market and with a potential growth at a CAGR exceeding 20% according to market analysts^7,8^. Typical Si photonics transceivers may include: modulators based on Si junctions realized by implantation doping^9^, modulators based on germanium (Ge) or silicon-germanium (SiGe) epitaxy^10,11^, photodetectors based on Ge epitaxy^12^.

State of the art Ge photodetectors integrated in a Si Photonics platform have featured 90 Gb s^−1^ data rate measured in a IM-DD (intensity modulation direct detection) optical signal^13^.

In the comparison with InP based detectors, the bandwidth limit of a waveguide integrated InP based detector for telecommunications is higher than the Ge case and it can reach, however, the complexity of the detector and the cost of the InP platform is not comparable with the cost structure of Si Photonics or graphene integrated in Si Photonics, and with the possibility of mass production offered by CMOS foundries.

Conversely 2D materials have recently emerged as viable alternative enablers for Si photonics devices^14^. In particular, graphene has been recognized as a promising material to be integrated on Si photonics to fulfill the requirements of next generation transceivers for datacom and telecom applications^15^. Graphene is an allotrope of carbon with atoms arranged on a one-atom thick hexagonal lattice^16^. The peculiar atomic structure, made of covalent bonds, determines a linear dispersion between energy and momentum with the conduction and valence bands meeting in single point (Dirac point) in the momentum space, with no energy gap^17^. This feature leads to intriguing electronic and optical properties, e.g., ultra-high carrier mobility^18^ and enhanced electric field effect^19^ that allows tuneable electronic and optical properties by electrical gating^19,20^. Moreover, graphene can be grown on a sacrificial material by chemical vapor deposition (CVD)^21^ and then transferred on top of any fully passive photonic waveguide platform, e.g., Si or silicon nitride (SiN), in order to realize broadband photonic integrated photodetectors^22^, fast electro-absorption^23^, and electro-refraction^24^ modulators, with a potential impact on the cost of future graphene photonic transceivers^15^.

Graphene photodetectors offer several advantages over the typical Ge photodetectors^12^. The first is the ultra-broadband optical absorption of graphene from the UV to the far infrared and beyond, which is due to the gapless band structure^25^. Moreover the fast dynamics of hot carriers (HCs) upon optical excitation, with relaxation times of the order of few ps^26–28^, enables optical to electrical conversion at bandwidths exceeding hundreds of GHz^29,30^.

Most of the devices demonstrated so far are based on the PB and PC effects, showing good current responsivity up to 0.5 AW^−1^ ^31^ and fast operation, however they operate under a bias voltage and significant dark direct current (DC) that can be in the order of several mA’s^31–35^. So high DC currents may introduce two main impairments in the design and implementation of a photoreceiver for optical communication, i.e., when we consider the circuit including the read-out electronics. The first is the noise arising from the photodetector. The noise analysis of graphene photodetector based on bolometric or photoconductive is typically not reported, and the effect of the large dark current on the signal integrity is not discussed. In general, those high DC currents may contribute to the overall noise in graphene devices^36,37^. The second impairment arises when we consider the coupling of the photodetector to the transimpedance amplifier (TIA), used to convert the photocurrent into a voltage signal to a level that can be processed by the electronics downstream of the TIA^38^. When the input DC components exceeds several mA the TIA may operate in a nonlinear regime, or may even saturate the output, introducing undesirable output offsets and reducing significantly the linear dynamic range needed to preserve the signal integrity^39^. Capacitive DC blocking is generally unfeasible at the input of TIAs because it requires large capacitors to preserve the signal, which are difficult to be integrated within the TIA electronics because of the large area required; moreover, the parasitic due to external DC blocks may deteriorate the TIA performance in terms of noise and bandwidth^39–41^. Depending on the application, the DC current may be canceled using specific cancellation circuits, differential inputs or balanced photodetection^38^. In the first case, high dark DC currents may still run the TIA into nonlinear regime because the cancellation is obtained with a feedback control after amplification^38^. In the case of differential input or balanced photodetection, large DC currents may give rise to an input DC component which may be still too high depending on the common mode rejection ratio (CMRR) of the differential amplifier, or on the balance of the photodetectors currents^38^. For these reasons, low dark DC currents are always desirable to improve the signal integrity and graphene photodetectors based on the PB or PC effects may introduce strong limitations to the TIA design. In photo-thermoelectric (PTE) based photodetectors, a photovoltage is generated through a thermoelectric effect induced by photon absorption^42^. PTE effect is highly efficient, as a large portion of the photon energy is transformed in electron heat owing to the ultrafast carrier scattering and weak coupling to phonons in graphene^27,43^. In particular, absorbed photons generate HCs with a carrier temperature spatially distributed along the graphene layer following the optical intensity distribution^44^. By inducing a spatial profile of the Seebeck coefficient in the material, e.g., by electrical gating, an electromotive force is generated by thermoelectric effect (Seebeck effect) which is proportional to the spatial gradient of the HC’s temperature^45–47^. The generated photovoltage does not require an externally applied bias, allowing zero dark current operation and direct voltage generation^46^. These properties are very appealing in terms of noise and power consumption, as PTE allows direct connection between the graphene photodetector and the read-out electronics without TIAs^15^ or allowing the use of a voltage amplifier. This is a potential breakthrough for this technology as the scalability of TIAs towards high frequencies (>50 GHz) is challenging, because of intrinsic design trade-offs^48^. In fact, the bandwidth of a TIA is inversely proportional to the feedback resistance, which sets the gain of the TIA. Increasing the bandwidth comes then in a tradeoff with the gain which should be high enough to amplify the signal and reduce the input referred noise current^38^. For these reasons the scalability of TIA’s towards high bandwidths is challenging. In fact, only a few >50 Gbit s^−1^ capable direct detection optical receivers or transimpedance amplifiers have been demonstrated to date^13^. In the case of our PTE 50 Ω-matched voltage photodetector, a linear low noise wide-bandwidth preamplifier can be used which is a technology already commercially available up to 66 GHz analog bandwidth.

Waveguide integrated graphene detectors based on PTE effect have been reported in recent years showing voltage responsivity in the range 3.5–28 V W^−1^ ^49–53^. These devices are based on the enhancement of the gradient of HC’s temperature through the improvement of the optical absorption by means of photonic structures confining the optical field in ~100 nm gaps^49,51,53^ or by exploiting the resonance of a microring resonator^50^. Despite the remarkable voltage responsivity^50,53^, detection of an optical data transmission using PTE-based graphene photodetectors operating in unbiased condition has not been demonstrated yet because of the high impedance of the devices. The optimization of the photovoltage signal in a PTE detector requires setting the detector at an operating condition near the charge neutrality point (CNP), i.e., where the conductivity of graphene is lowest. For this reason, typical PTE photodetectors exhibit output resistance from several hundreds to thousands of Ohms^49–51,53^, i.e., too high for proper matching to the typical 50 Ω impedance of the test instruments.

Here we report on a PTE graphene photodetector integrated on a Si photonic waveguide having a voltage responsivity of about 3.5 V W^−1^ and a 3 dB bandwidth of 70 GHz, limited only by the instrumentation, showing no roll off in the measured range. We optimized the design of the PTE detector to match 50 Ω resistance to be suitable for data communication system tests. We demonstrate the detection of high baud rate optical data stream, in absence of dark current, by testing our device up to 105 GBaud Non-Return-to-Zero (NRZ) On-Off-Keying (OOK) and 60 GBaud 4-level-Pulse-Amplitude-Modulation (PAM4) for a net bit rate of 120 Gb s^−1^.

The relevance of this demonstration is that graphene is a material that can be easily integrated on Si Photonics waveguide and it has the advantage with respect to Ge used for Si Photonics detectors, to scale in speed. In fact the highest data rate measured with a Ge detector in a Si photonics waveguide is 90 Gb s^−1^ with a NRZ optical whereas in this work the detected NRZ signal was at 105 Gb s^−1^ and 120 Gb s^−1^ with PAM4 modulation format. Moreover the present data rate measurements could be extended to higher speed because a direct measurement of the graphene photodetector bandwidth showed a value of at least 128 GHz^32^ and, from another experimental estimation greater than 270 GHz^30^. These values indicate the potentiality of graphene in Si Photonics to scale beyond the limits of Ge detectors.

## Results

### Device concept and simulations

**Fig. 1:**
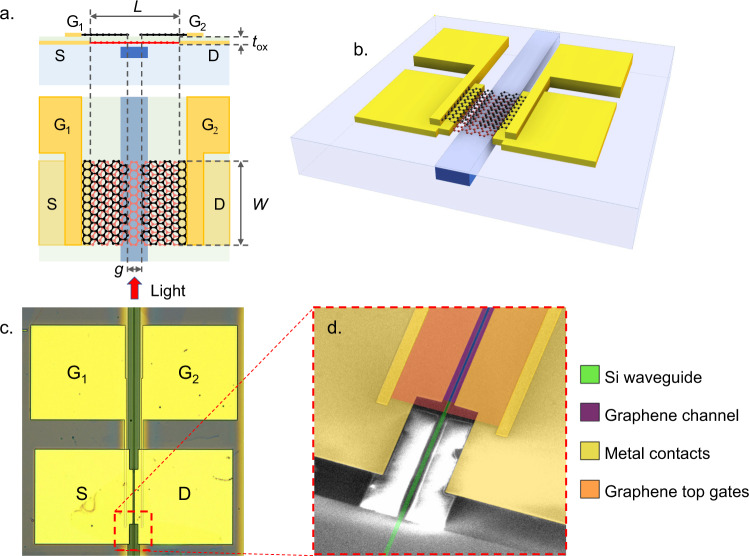
Structure of the realized device. **a** Cross section and top view of the designed PTE graphene photodetector. The active region is the first layer of graphene: the channel (red) of length *L* and width *W*, on top of the 480 nm × 220 nm Si waveguide (blue, light blue is SiO_2_). The channel is accessed electrically by the source (S) and drain (D) metal contacts (yellow). A 100 nm thin SiN layer (light green) is used as dielectric spacer for the gates. Top gates are realized with a second layer of graphene (black) patterned in a split gate configuration with a gap (g) of 150 nm. Graphene gates are contacted through metal contacts (G_1_ and G_2_). **b** 3D schematic of the proposed PTE graphene photodetector. **c** Optical microscope picture of the fabricated devices: scale bar is 20 µm. **d** False color SEM image of the input of the fabricated PTE graphene photodetector: green the Si waveguide, yellow the metal contacts, purple the active graphene channel (violet on the Si waveguide), orange the second graphene gates. Scale bar is 5 µm.

We designed a waveguide integrated PTE photodetector based on a split-gate geometry similar to the one used in a previous work^54,55^ (Fig. 1).

In refs. ^54,55^ we demonstrated the high speed of operation the PTE detector integrated on a SiN waveguide (core cross-section 1200 nm × 260 nm) using poly-vinyl alcohol (PVA) as gate dielectric. The use of PVA allowed to preserve the good quality of graphene after polymer deposition but, at the same time, posed severe limitations: large hysteresis of graphene field effect curves^56^, contact resistance larger than 5000 Ω µm, and lithographic limitations as discussed in ref. ^54^. In this work, we moved to the SOI platform to exploit the steeper mode profile of the Si waveguides, which is due to the higher index contrast with respect to SiN. The steeper mode profile corresponds to a steeper profile of the temperature of the hot electrons, i.e., higher derivative and by consequence higher responsivity (see Supplementary Note 1–3). Moreover, we used SiN rather than PVA because SiN provides better performance in terms of dielectric strength, hysteresis, and lithographic limitations^23^. This technology platform, i.e., double layer graphene separated by SiN dielectric on SOI, was developed by some of us for the realization of graphene on Si optical modulators^23^. It is characterized by a low hysteresis and low contact resistance (>500 Ω µm), which is an important parameter in order to match the 50 Ω impedance target.

The photodetector has been integrated on top of a transverse electric (TE) silicon on insulator (SOI) waveguide designed for single mode operation at 1550 nm wavelength, with core cross section 480 nm × 220 nm (Fig. 1a). The top cladding is thinned and planarized to a final thickness of about 20 nm on the top of the waveguide in order to maximize the interaction of the evanescent mode field with the graphene layer transferred on top of the silicon core (see “Methods” section). The detector consists of a stack of two layers of graphene separated by a *t*_ox_ = 100 nm thick SiN layer (Fig. 1a). The photo-conversion occurs in the first single layer graphene where optical absorption of light generates a gradient of HCs’ temperature (see Supplementary Note 1). The second layer of graphene is used to realize two split gates (G_1_ and G_2_) separated by a 150 nm large gap (g in Fig. 1a), used to electrostatically induce a step change of the Seebeck coefficient in the first graphene layer. We refer to the distance between the source (S) and drain (D) metal contacts as the channel length *L* and to the width of the first graphene as the channel width W (Fig. 1a). The generated photovoltage can be approximated as^45^:

1 $$V_{{\mathrm{ph}}} = \left( {S_1 - S_2} \right)\Delta T,$$

where *S*_1_ and *S*_2_ are the Seebeck coefficients of graphene below gate 1 and gate 2 (Fig. 1a), $\Delta T = \left( {\bar T_{{\mathrm{HC}}} - T_0} \right)$ is the difference between of the HCs’ temperature averaged over the graphene channel ($\bar T_{{\mathrm{HC}}}$), which is proportional to the absorbed optical intensity, and the graphene lattice temperature ($T_0$). The detailed model for the calculation of the photovoltage is in the Supplementary Information (see Supplementary Eqs. 1, 2). We remark that the magnitude of the Seebeck coefficient depends on graphene electronic properties, e.g., mobility and residual carrier concentration at the charge neutrality point, n*^45,57,58^. High quality graphene, i.e., having high mobility and low n*, exhibits higher Seebeck coefficient. e.g., experimental values <20 µV K^−1^ for polycrystalline CVD graphene (grain size <5 µm, mobility <1000 cm^2^ V^−1^ s^−1^), have been reported^59^, while high quality exfoliated graphene on hexagonal boron nitride (hBN) substrate (mobility > 10,000 cm^2^ V^−1^ s^−1^) has been demonstrated to exhibit *S* = 183 µV K^−1^ ^60^. In this work, we used a CVD grown single crystal graphene with mobility as high as 130,000 cm^2^ V^−1^ s^−1^ at room temperature when transferred on hBN substrates^61,62^. A single crystal graphene channel is important not only for the high Seebeck coefficient, but also for the absence of grain boundaries that may be responsible for non-uniform control of the Seebeck coefficient along the graphene channel^63^.

The device geometry shown in Fig. 1 has been optimized to maximize the photovoltage while matching the impedance between the device and the read-out electronics, e.g., a digital sampling oscilloscope (DSO) or a discrete radio frequency (RF) voltage amplifier. We modeled the photodetector as a voltage source with a series impedance given by both the channel sheet resistance (*R*_ch_) and the contact resistance (*R*_c_) (see Supplementary Note 5). For the sake of simplicity, we neglected the capacitive contribution arising from contacts and gates as they do not affect the impedance of the device within its bandwidth frequency range (see Supplementary Fig. 9). In this condition, the detector impedance is the geometrical resistance of the graphene channel, i.e., the series resistance *R*_pd_ = *R*_ch_*LW*^−1^ + 2*R*_c_*W*^−1^. Assuming a generated voltage *V*_ph_ = *R*_V_*P*_av_, where *R*_V_ is the voltage responsivity and *P*_av_ is the average power of a sinusoidal optical signal, we can write the electrical power *P*_L_ transferred to the load *Z*_L_ (50 Ω) as^64^:

2 $$P_{\rm{L}}\,=\,\frac{1}{2}V_{{\mathrm{ph}}}^2\frac{{Z_{\rm{L}}}}{{\left( {R_{{\mathrm{pd}}} + Z_{\rm{L}}} \right)^2}} = \frac{1}{2}R_{\rm{V}}^2P_{{\mathrm{av}}}^2\frac{{Z_{\rm{L}}}}{{\left( {\frac{{2R_{\rm{c}}}}{W} + \frac{{R_{{\mathrm{ch}}}L}}{W} + Z_{\rm{L}}} \right)^2}},$$

Equation (2) shows that the optimization of the power transfer is obtained through the optimization of the device length and width. As these parameters affect also the voltage responsivity, we tailored the mathematical model proposed by Song et al.^46^ to the case of a waveguide integrated photodetector (see Supplementary Note 1).

**Fig. 2:**
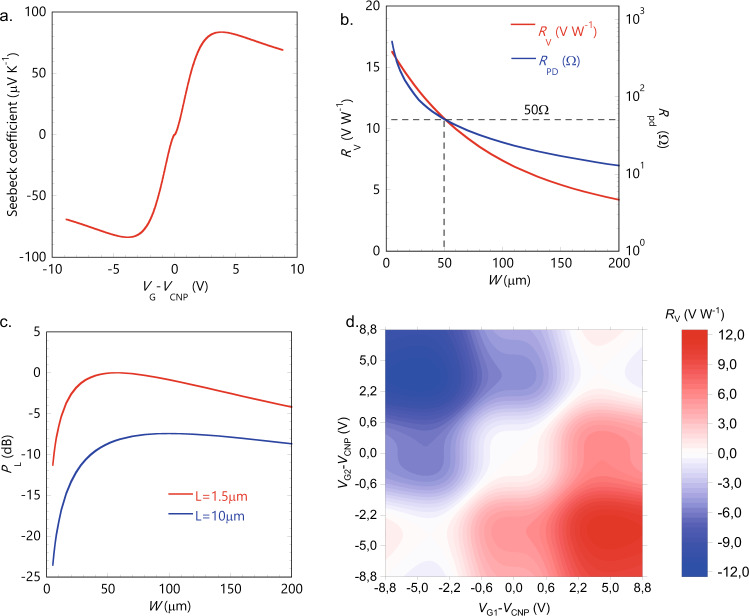
Simulation results. **a** Seebeck coefficient as a function of the gate voltage *V*_G_, shifted by *V*_CNP_, that is the the charge neutrality voltage value. **b** Voltage responsivity *R*_V_ (red curve) and device resistance *R*_pd_ (blue curve) versus the channel width *W*. **c** Normalized electrical power *P*_L_ transferred from the detector to the input impedance of the read-out electronics (*Z*_L_ = 50 Ω) as a function of the channel width *W*. We report the case of a channel length *L* = 1.5 µm (red curve) and channel length *L* = 10 µm (blue curve). The two curves are normalized to the maximum transferred power in the case of *L* = 1.5 µm. **d** Six-fold voltage responsivity *R*_V_ map of the designed PTE photodetector with *L* = 1.5 µm and *W* = 50 µm.

In order to find the maximum of the photovoltage, we calculated the Seebeck coefficient of the device as a function of the gate voltage, i.e., chemical potential *µ*_c_ (Fig. 2a) (see Supplementary Note 2). For the simulations, we assumed the graphene sheet and contact resistances as measured in a previous work^23^ were the same fabrication process has been exploited. More specifically, we used *R*_C_ = 500 Ω µm and *R*_ch_ = 1000 Ω/sq at a chemical potential of ~180 meV, very close to the maximum responsivity point (*µ*_C_ = ±130 meV). We simulated the DC behavior by setting *µ*_c_ = ±130 meV, corresponding to the maximum (positive) Seebeck coefficient on one side of the junction and to the minimum (negative) on the other side, in order to simulate the maximum voltage responsivity *R*_V_. Then we swept the channel dimensions *L* and *W* to study how they affect the maximum *R*_V_. In particular, the channel length *L* has a minimum impact on the generated photovoltage, as long as it is larger than the optical mode width in the transverse direction (see Supplementary Note 4). We set a fixed *L* = 1.5 μm which is the minimum distance between side metal contacts to avoid optical losses, as predicted by simulations (see Supplementary Note 3 and Supplementary Fig. 5). On the contrary, the channel width *W* affects both the device resistance and the voltage responsivity as it determines the maximum propagation length of light in the graphene channel, i.e., the light absorption region (see Fig. 1). In Fig. 2b, we show the evaluated maximum responsivity (red curve) versus channel width *W* in the range 5–200 μm and the corresponding device resistance *R*_pd_ (blue curve)_._ The voltage responsivity *R*_V_ decreases with the device width *W*, reaching half of its maximum at about *W* = 100 μm (Fig. 2b). This is due to the exponential decay of the optical intensity in the propagation direction caused by the absorption in the graphene layer (see Supplementary Note 4 and Supplementary Fig. 6). The device resistance *R*_pd_ decreases at increasing channel width and reaches 50 Ω for *W* = 50 μm. The optimal condition for electrical power transfer from the detector to the load is obtained for a device width in the range 50–70 µm, depending on the channel length *L* (Fig. 2c). For shorter *W*, *P*_L_ drops because of the larger series resistance of the photodetector, while longer W limits the power transfer because of the voltage responsivity drop. The result is an optimum geometry with *L* = 1.5 μm and *W* = 50 µm. In Fig. 2c we compare *P*_L_ for devices with channel length *L* = 1.5 μm and *L* = 10 μm normalized to the maximum transferred power obtained for *L* = 1.5 μm. In the non-optimized case *L* = 10 μm, the device resistance is larger (220 Ω), causing a worse impedance matching resulting in a lower maximum power transfer (*P*_L_^MAX^ ≈ −7 dB). Figure 2d shows the calculated voltage responsivity map as a function of the two gate voltages *V*_G1_ and *V*_G2_ (with source drain voltage *V*_SD_ = 0 V), for the optimum design (*L* = 1.5 μm). The responsivity map shows the expected PTE six-fold pattern with an absolute maximum of ~10 V W^−1^ in the np and pn regions, for effective gate voltages *V*_G1_ − *V*_CNP_ = −5V, *V*_G2_ − *V*_CNP_ = 5 V (np region), and *V*_G1_ − *V*_CNP_ = 5 V, *V*_G2_ − *V*_CNP_ = −5V (pn region), respectively.

We fabricated the device on a standard SOI platform with 220 nm thick Si overlayer and 2 μm thick buried oxide (BOX) (see “Methods” section). We used high quality single crystal graphene grown by chemical vapor deposition (CVD) and transferred on the Si waveguides by a semi-dry transfer technique demonstrated previously^62^. After the transfer and patterning of the first layer of graphene, a stack of Nickel (Ni) and Gold (Au) was used to realize the source and drain metal contacts. Then, we transferred single-layer of hBN to protect the first layer graphene from the subsequent plasma-enhanced chemical vapor deposition (PECVD) of 100 nm of SiN used as gate dielectric. The second graphene layer was grown, transferred, and patterned using the same procedures utilized for the first layer. Top gate contacts were deposited using the same process as for the first graphene channel. We used Raman spectroscopy^65^ to characterize the two graphene crystals (see “Methods” section).

### DC characterization

**Fig. 3:**
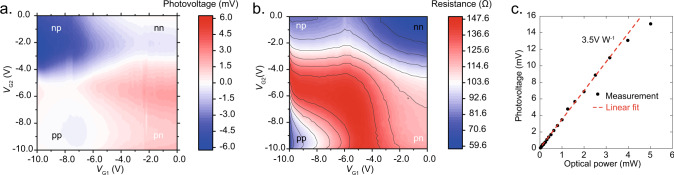
DC characterization. **a** Measured photovoltage map of the fabricated PTE graphene photodetector (*L* = 1.5 µm and *W* = 50 μm) as a function of the gate voltages *V*_G1_ and *V*_G2_. Optical input was ~1.65 mW at the detector input facet. **b** Measured resistance map of the fabricated PTE graphene photodetector (*L* = 1.5 µm and *W* = 50 μm) as a function of the gate voltages *V*_G1_ and *V*_G2_. **c** Photovoltage as a function of the optical power at the detector input facet. Black dots are experimental values, the red dashed line represents the linear fit.

We have experimentally characterized the static behavior of the PTE photodetector by mapping the photovoltage (Fig. 3a) and the resistance (Fig. 3b) as a function of the applied gate voltages. We coupled the light of a continuous wave (CW) laser source at 1550 nm wavelength into the SOI waveguide by means of a single mode optical fiber and a single polarization grating coupler (see “Methods” section). We swept the bias applied to both the gate electrodes with respect to the channel source electrode between 0 V and −10 V and measured the photovoltage imposing zero current between the source and drain electrodes (see “Methods” section). The device resistance map (Fig. 3b) has been obtained in a similar way measuring the resistance of the detector for each couple of gate voltages in the absence of the optical excitation.

The photovoltage map clearly shows different regions where the photovoltage changes its sign, which is the signature of the dominance of PTE over other photoconversion mechanisms like photovoltaics at zero bias^46^. We show a photovoltage map having a maximum absolute value of about 6 mV for an optical input power at the detector equal to 1.65 mW (see Methods), which results in a voltage responsivity of about 3.5 V W^−1^ for *V*_G1_ = −1 V and *V*_G2_ = −8 V. The asymmetric photovoltage map may be explained by an asymmetry in the energy dependence of the carrier relaxation time in graphene between the n-type and p-type transport^60^. This behavior leads to an asymmetric Seebeck coefficient profile^60^ with respect to the charge neutrality point. The device resistance in proximity of the maximum responsivity is about 90 Ω, which is higher than the designed 50 Ω. The difference can be ascribed to deviations in the sheet and contact resistances of the fabricated device with respect to the design. We measured the linearity of the photovoltage as a function of the input optical power (Fig. 3c) (see “Methods” section). The photoresponse is linear up to 3 mW, with the slope corresponding to a voltage responsivity of 3.5 V W^−1^. We argue that the low responsivity is probably due to the first graphene layer whose quality is deteriorated by the PECVD process deposition of the 100 nm thick SiN (see Raman characterization in “Methods” section). This is the main difference with respect to the previously reported process developed for the fabrication of graphene modulators^23^, for which a SiN thickness of ~20 nm has been used between the two graphene layers. Although the first graphene layer (i.e., the channel layer) is protected by hBN to preserve the material quality, the process needs further improvements. An improved encapsulation^66^ would give results closer to what is predicted by our simulation (see Supplementary Note 4). Moreover, the responsivity may be further increased by adopting optical absorption enhancement, as demonstrated in refs. ^50,53^.

### Detection of optical data streams

Although the device resistance was larger than the designed 50 Ω, we used the fabricated PTE photodetector to demonstrate the detection of high baud rate optical data streams. In a first set of experiments, we used the device to detect a Non-Return-to-Zero (NRZ) On-off-Keying (OOK) optically modulated signal at 28 Gb s^−1^ (see “Methods” section). PTE operation does not require an applied voltage between the source and drain electrode (bias-free). We used a bias-tee to monitor the DC component of the photovoltage and to optimize the responsivity (see “Methods” section). We found the maximum responsivity (~3 V W^−1^) for gate voltages close to the ones of the maximum *R*_V_ in the DC map, i.e., *V*_G1_ = −1 V and *V*_G2_ = −8 V.

**Fig. 4:**
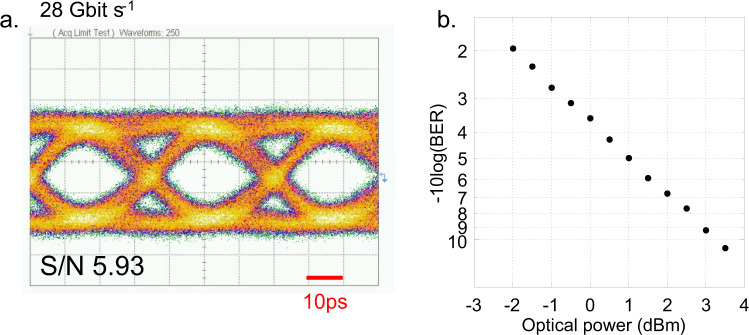
Detection of an optical data stream at 28 Gb s^−1^. **a** Eye diagram collected for a 28 Gb s^−1^ NRZ OOK data stream at the input of the PTE graphene photodetector. The optical power at the input facet of the photodetector was 2 mW. **b** BER curve as a function of the optical power at the input facet of the detector. In both measurements, an electrical RF amplifier with 23 dB gain and 35 GHz bandwidth has been used.

A 50 Ω matched commercial RF amplifier with 23 dB gain and 35 GHz bandwidth has been used to amplify the electrical signal generated by the photodetector. Figure 4 shows the collected eye diagram and corresponding bit error ratio (BER) curve as a function of the optical power at the detector input (see “Methods” section). A BER equal to 1.8 × 10^−11^ has been obtained for an input optical power of 3.5 dBm in back-to-back configuration, i.e., by connecting through a short fiber (few meters) the detector to the transmitter.

**Fig. 5:**
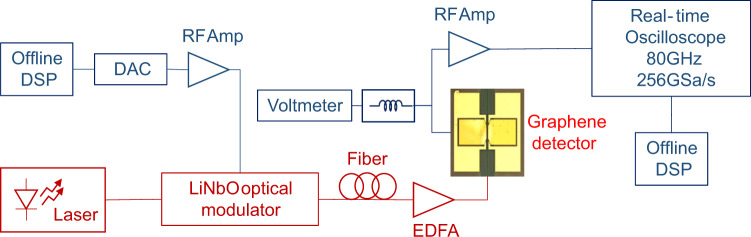
Experimental setup. High speed optical testbed with a DAC based transmitter and an ADC based receiver.

In order to test our graphene detector at higher baud rates, we performed a second set of experiments involving a digital-to-analog converter (DAC) based optical transmitter with digital pre-emphasis. This is used to compensate for DAC and driver losses and for the limited bandwidth of the LiNbO_3_ Mach Zehnder modulator (MZM) used to modulate the optical CW signal (see “Methods” section). After back-to-back transmission the output voltage of the detector is amplified using a commercial amplifier with 22 dB gain and 65 GHz bandwidth. A real time oscilloscope and offline digital signal processing (DSP) is used for data acquisition, signal recovery and performance assessment. A block diagram of the setup is shown in Fig. 5.

**Fig. 6:**
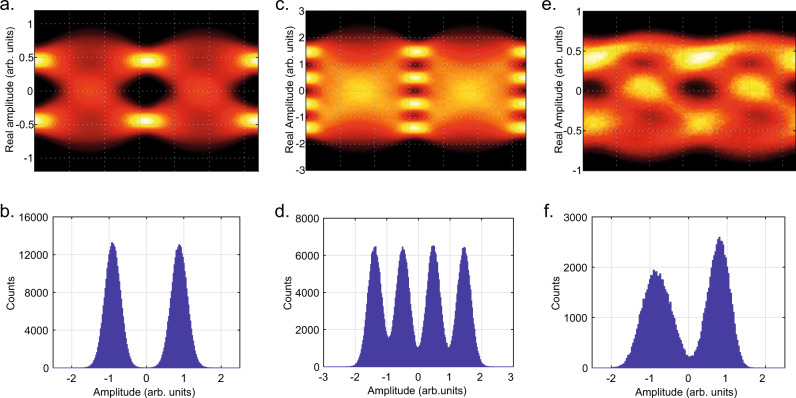
Detection of an optical data stream at 60 GBaud and 105 GBaud. Eye diagrams collected for a 60 GBaud NRZ OOK (**a**), 60 GBaud PAM4 (**c**), and 105 GBaud NRZ OOK (**e**) data stream at the input of the detector. Histograms of the signal amplitude collected at the optimum sampling point for a 60 GBaud NRZ OOK (**b**), 60 GBaud PAM4 (**d**), and 105 GBaud NRZ OOK (**f**) data stream at the input of the detector.

We tested our device at 60 GBaud using an NRZ OOK and a four level Pulse Amplitude Modulation (PAM4) format, and at 105 GBaud NRZ OOK. The collected eye diagrams and the corresponding histograms of the signal amplitude at the optimum sampling point are shown in Fig. 6a–d.

The evaluated SNR values of the collected data are 12.5 dB for the NRZ data transmission and 13.7 dB for the PAM4. The corresponding BER is 8 × 10^−5^ (NRZ) and 1.1 × 10^−2^ (PAM4). In order to show the high bandwidth of the device, we repeated the experiment up to 105 GBaud NRZ data transmission. The collected eye diagram (Fig. 6e) exhibits SNR = 8.4 dB with a resulting BER of 8 × 10^−3^. The associated signal amplitude at the optimum sampling point (Fig. 6f) shows an asymmetry towards the zero level because of a non-optimized signal at the transmitter side.

### RF characterization

**Fig. 7:**
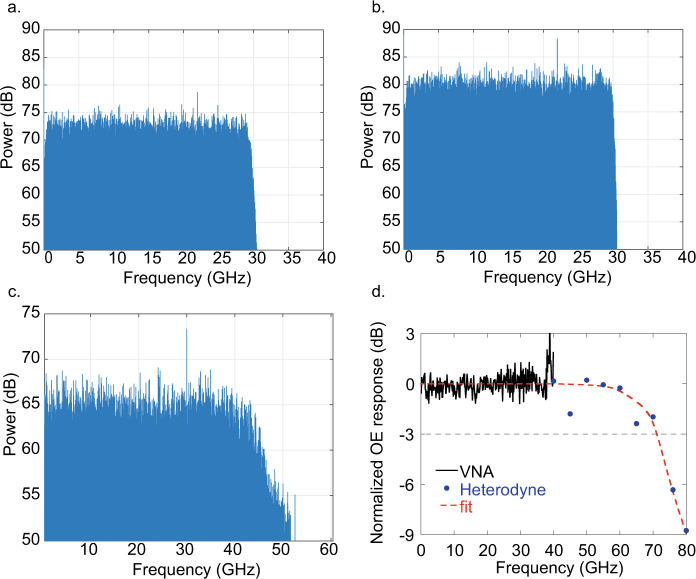
Frequency response. Electrical spectra of the received signals: 60 GBaud NRZ OOK (**a**), 60 GBaud PAM4 (**b**), and 105 GBaud NRZ OOK (**c**). **d** Optical to electrical bandwidth of the received signal: black curve is the measurement as acquired from the VNA, blue dots are the measured response as obtained by the heterodyne setup with laser beating, the red dashed line is the interpolation curve.

In Fig. 7a–c we show the electrical spectra of the received signals of Fig. 6 obtained through a fast Fourier transform (FFT) of the real time signal after the equalization filter (see “Methods” section).

The spectra are completely flat in the range of frequencies of the input electrical signal as generated at the transmitter side without any ripples or a drop at high frequencies. This demonstrates the high speed of operation of our detectors, as already demonstrated for a similar device in our previous work where we showed flat response up to 40 GHz^54,55^. The response shown in Fig. 7c. is flat up to 40 GHz, beyond this we see a 10 dB drop of the power at signals Nyquist frequency. In order to understand if it is related to the photodetector, we investigated the flatness of the frequency response by means of a Vector Network Analyzer (VNA, see “Methods” section) up to 40 GHz, which is shown in Fig. 7d. The peak close to 40 GHz is ascribed to the electrical setup. The frequency response above 40 GHz up to 80 GHz was then measured by implementing a heterodyne setup superimposing two CW tunable lasers having frequencies *f*_1_ and *f*_2_, which are coupled through a 3 dB fiber coupler and interfere causing a response at the beating frequency *f*_beating_ = *f*_2_ − *f*_1_. We set *f*_1_ constant and varied *f*_2_ in order to have *f*_beating_ in the range 40–80 GHz at steps of 5 GHz. We obtained the frequency response by numerically computing the FFT of the acquired real time traces and extracting the peak intensity of the signal in the frequency domain. Results are shown as blue dots in Fig. 7d. The frequency response is almost flat up to 60 GHz, with a drop of −2 dB at 45 GHz, most probably due to the experimental setup. In fact, this drop is much lower than what observed in the spectrum of Fig. 7c, and it is fully recovered at 50 GHz. Similar drop is observed at 65 GHz, while at 70 GHz the curve is still above the −3 dB line and then start decreasing because of the roll-off of the used RF amplifier (SHF804B specified flat up to 60 GHz). From the extracted frequency response, we can conclude that the drop shown in Fig. 7c is not arising from the graphene photodetector, while it is limited by the RF amplifier and in general by the setup. The origin of the roll-off will be investigated with further experiments.

In conclusion, we demonstrated a waveguide integrated graphene photodetector based on the PTE effect operating at zero bias without dark current. The device exhibits a responsivity of about 3.5 V W^−1^ with a flat frequency response up to 65 GHz. In this work we demonstrated the detection of an optical data transmission by using different modulation formats at baud rates up to 105 GBaud, by using the PTE effect in unbiased operation. This work demonstrates the feasibility of using PTE based graphene detectors as optical receivers and paves the way for the realization of versatile and highly efficient optical graphene transceivers.

## Methods

### Fabrication

We used electron beam lithography (EBL) to realize single mode transverse electric (TE) polarized Si photonic waveguides with cross section 480 × 220 nm on a standard SOI substrate with 220 nm thick Si overlayer and 2 μm thick BOX. The waveguide is provided with single polarization grating couplers designed to couple the light to the fundamental TE mode of the Si waveguide. The waveguides were then covered with a with a thin layer of tetraethyl orthosilicate (TEOS) and a thick boron-phosphorus TEOS (BPTEOS) cladding which was next thinned down to a final thickness of ~25 nm on top of the waveguide. Graphene was grown on copper (Cu) foils by deterministic seeded growth by chemical vapor deposition (CVD)^62^. We used Chromium (Cr) nucleation seeds the Cu foils and patterned by optical lithography. Regular arrays of graphene crystals were grown at 1060 °C with background pressure of 25 mbar using a 4″ BM Pro cold-wall reactor. Sample enclosure and Argon (Ar) annealing were used to control the nucleation density^62^. After growth, a PMMA layer was spin-coated on the Cu foil and an adhesive frame was attached to the perimeter of the sample. Electrochemical delamination of the graphene crystals was used to separate graphene from the Cu growth substrate by applying a voltage of −2.4 V with respect to a Cu counter-electrode in a 1 M NaOH solution. The delaminated graphene array on the PMMA membrane was thoroughly rinsed in deionized water. We then aligned the array precisely to the target waveguides using a micrometric 4-axis stage and laminated it at 90 °C. The sample was heated at 105 °C for 5 min to improve the adhesion. The PMMA support was then removed in acetone. EBL and reactive ion etching (RIE) (5 sccm oxygen and 80 sccm argon, 35 W power) were used to pattern graphene by using PMMA as an etch mask. After the transfer and patterning of the first graphene layer, metal has been thermally evaporated to realize top contacts. We used a stack 7 nm of Ni and 60 nm of Au, followed by lift-off in acetone. The fabricated graphene channel was next coated with a protective layer of a commercially available large-area polycrystalline single-layer hBN provided by Graphene Laboratories Inc. hBN was used to provide protection to the first layer graphene with respect to the plasma used during the subsequent PECVD (350 °C) of the 100 nm SiN dielectric film. The second graphene layer used as gates was grown, transferred and patterned with the same process described for the first layer. Finally, metal contacts to the graphene gates have been realized with the same thermal evaporation of Ni and Au.

### Raman characterization

Raman spectroscopy has been used to characterize the quality of the SLGs used for photodetectors fabrication. All the Raman spectra have been collected in a Renishaw InVia spectrometer, equipped with a 532 nm excitation laser, at laser power ~1 mW and acquisition time of 4 s. The measurements have been performed for each fabrication step. Starting from pure transferred graphene, the Raman spectra has a 2D peak with a single Lorentzian shape and FWHM(2D) ∼25 cm^−1^, showing a low level of sub-µm strain fluctuations (and thus relatively high carrier mobility)^67^, comparable to exfoliated graphene on SiO_2_. Due to subsequent hBN transfer and SiN deposition an increase of the FWHM(2D) peak is reported, obtaining ∼25.5 and ∼32.5 cm^−1^, respectively, indicating an increase in strain fluctuations and thus lower carrier mobility.

### DC measurements

Static characterization of the graphene photodetector has been performed by using two electrical source-meters: the first was used to sweep the gate voltages with two independent channels, the second was used to set an open circuit (zero current) between source and drain and to read the generated photovoltage. A ground-signal (GS) probe was used to probe the source and drain, while DC needle probes were used to contact the gates. A CW laser source was amplified by an Erbium-doped fiber amplifier (EDFA) and coupled to the chip with a single mode optical fiber coupled to the input grating coupler. A fiber-based polarization controller was used to match the required polarization at the input grating coupler. After the input grating coupler, we used an integrated 3 dB splitter: one output branch of the splitter was coupled to the PTE photodetector, while the other branch was used to monitor the optical power through an output grating coupler. After the PTE photodetector, another grating coupler was used to monitor the optical power at the device output. In this way we could perform a precise characterization of the insertion loss of the device and properly evaluate the optical power at the device input. We estimated a total insertion loss of −13 dB due to the grating coupler efficiency (−5 dB), the 3 dB splitter (−3 dB) and propagation loss in the access waveguide (−5 dB). This last is due to particle (polymers and metal) deposition on the access waveguide which is not protected with cladding. Optical power dependent measurements were done by using a fiber-based variable optical attenuator (VOA) after the EDFA.

### Broad band measurement

The first set of measurements was performed by using a commercial pattern generator and BER tester at 28 Gb s^−1^. A LiNbO_3_ MZM having 30 GHz bandwidth was used to modulate the optical signal. A variable optical attenuator (VOA) was used to perform the BER measurements as a function of the input optical power. A 50 Ω matched commercial RF amplifier with 23 dB gain and 35 GHz bandwidth was used to bring the electrically generated signal to a level readable by a digital sampling oscilloscope (DSO) and by the BER tester.

The chip level Graphene photodetector was interconnected on a probe station with the same fiber coupling scheme described above. For RF interconnection we used a 67 ground-signal (GS) probe to access the electrical signal generated by the photodetector. A 67 GHz bandwidth bias-tee was used to impose zero DC current to the device.

We used the same MZM of the previous setup for the measurement of the frequency response. Port A of the VNA was used to drive the MZM and the second port to measure the scattering parameter S21 at the output of the graphene photodetector. We used a commercial 70 GHz bandwidth photodetector to measure the response of the setup, i.e., modulator, cables, probes, and connectors. The electrical amplifier was not used for the frequency response measurement.

The second set of measurements was performed at higher speed. In the transmitter we used an 88 GSa s^−1^ DAC to generate the PAM data. Pre-calculated data were sent out periodically. In the transmitter digital signal processor, we applied raised cosine pulse shaping and in addition we compensated for the DAC, driver amplifier and modulator low pass characteristics. The electrical signal is modulated onto a 1550 nm optical carrier supplied by an external cavity laser, the modulator was biased at quadrature. The optical signal was amplified using an EDFA before launching onto the photodetector. The electrical response of the device is amplified using a 65 GHz bandwidth amplifier having 22 dB gain. Next, we digitized the data in a real time oscilloscope at 33 GHz bandwidth and stored the data for subsequent digital signal processing (DSP). The offline DSP incorporates resampling to 2 sample/symbol to simplify filter implementation. Next, we applied the channel compensation using a linear feed forward equalizer, where the filter taps where adapted respectively. Finally, we assessed the performance by counting the bit errors to determine the bit error ratio (BER) as well as the signal to noise ratio (SNR).

In a third experiment we further increased the speed by replacing the DAC and ADC. In the transmitter we applied a 120 GSa s^−1^ DAC and generated NRZ data at 105 GBaud. The data where amplified in a 65 GHz driver and modulated using LiNbO_3_ modulator. In the receiver we digitized the data at 256 GSa s^−1^ and 80 GHz bandwidth. Further details were identical to the second experiment.

## Supplementary information

Supplementary Information

## Data Availability

The data that support the findings of this study are available from the authors on reasonable request, see author contributions for specific data sets.
