# Supplementary material for: Photo thermal effect graphene detector featuring 105 Gbit s−1 NRZ and 120 Gbit s−1 PAM4 direct detection
Source: Nat Commun. 2021 Feb 5;12:806. doi: 10.1038/s41467-021-21137-z (PMC7864989; doi:10.1038/s41467-021-21137-z)
Supplement: Supplementary file 1 — Supplementary Information [file 41467_2021_21137_MOESM1_ESM.pdf]

Supplementary Material to

Photo Thermal Effect Graphene Detector Featuring 105 Gbit s^{-1} NRZ and 120 Gbit s^{-1} PAM4 Direct Detection

S. Marconi[1], M. A. Giambra[2], A. Montanaro[2], V. Mišeikis[3,4], S. Soresi[2,5], S. Tirelli[2,5], P. Galli[6], F. Buchali[7], W. Templ[7], C. Coletti[3,4], V. Sorianello[2] and M. Romagnoli[2,*]

[1]Tecip Institute – Scuola Superiore Sant'Anna, Via G. Moruzzi 1, 56124 Pisa, Italy

[2]Photonic Networks and Technologies Lab – CNIT, Via G. Moruzzi 1, 56124 Pisa, Italy

[3]Center for Nanotechnology Innovation @NEST - Istituto Italiano di Tecnologia, Piazza San Silvestro 12, I-56127 Pisa, Italy

[4]Graphene Labs, Istituto Italiano di Tecnologia, Via Morego 30, 16163 Genova, Italy

[5]Fondazione INPHOTEC, Via G. Moruzzi 1, 56124 Pisa, Italy

[6]Nokia Solutions and Networks Italia, via Energy Park 14, 20871 Vimercate, Italy

[7]Nokia Bell Labs, Lorenzstr. 10, 70435 Stuttgart, Germany

*corresponding author: marco.romagnoli@cnit.it

Supplementary Note 1 - Mathematical model of PTE based waveguide integrated photodetector

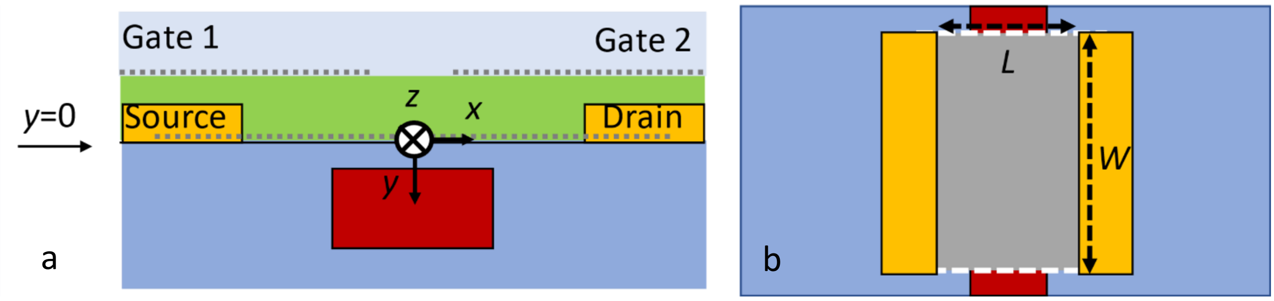

Supplementary Figure 1. Structure of the device. a) Cross section of the realized device. b) In plane cut of the active graphene layer, (x,z) plane at y=0.

The mathematical model used in the main text to compute the photovoltage and the voltage responsivity R_V is summarized by the set of Supplementary Equations 1.

$$\nabla \cdot (k_{\mathrm{HC}} \nabla T_{\mathrm{HC}}) + \frac{k_{\mathrm{HC}}}{L_{\mathrm{c}}^2} (T_{\mathrm{HC}} - T_{\mathrm{lattice}}) = \eta \frac{\mathrm{P_{in}}(x) \exp\left(-\frac{z}{L_a}\right)}{L_{\mathrm{a}}} - \mathbf{J} \cdot \nabla \Pi \qquad \text{(Supplementary Equation 1.a)}$$

$$\mathbf{J} = \sigma(-\nabla V + S \nabla T_{\mathrm{HC}}) \qquad \text{(Supplementary Equation 1.b)}$$

$$\nabla \cdot \mathbf{J} = 0 \qquad \text{(Supplementary Equation 1.c)}$$

All the parameters and variables are defined in the channel plane (active graphene layer, (x,z) plane in y=0, Supplementary Figure 1a-b). Eq. s1.a is the heat transport equation as defined in the work by Song et al.[1], which relates the absorbed optical power density to the hot carriers' (HCs) temperature. Supplementary Equation 1.b describes the current density $\mathbf{J}(x, z)$ in the graphene channel in the HCs regime accounting for photo-thermoelectric (PTE) contribution. Supplementary Equation 1.c is the continuity equation in stationary condition. The operator $\nabla = \left(\frac{\partial}{\partial \mathrm{x}}, \frac{\partial}{\partial \mathrm{z}}\right)$ is defined in the plane y=0 and acts on the x and z coordinates. $T_{\mathrm{HC}}(x,z)$ and T_{lattice} are the HCs and lattice temperature while $V(x,z)$ is the electrical potential in the active graphene channel. S and k_{HC} are the Seebeck coefficient and the HCs thermal conductivity in graphene, respectively. The Peltier term is computed as $\Pi = ST$ [1,2]. The term $(1/L_{\mathrm{c}}^2)\,(T_{\mathrm{HC}} - T_{\mathrm{lattice}})$ is the term associated to the cooling of HCs and L_{c} is the parameter known as cooling lenght[1]. We assumed L_{c} = 140 nm as reported in ref.[3] for polycrystalline graphene. $\eta \mathrm{P_{in}}(x) \exp(-z/L_{\mathrm{a}})/L_{\mathrm{a}}$ is the source term, i.e., the optical power density absorbed at coordinate z (along the light propagation direction) in a point of the graphene channel (y=0) and therefore delivered to the hot carriers system. L_{a} is the absorption length of the optical mode and η is the fraction of the absorbed optical power which is absorbed in the channel layer and not by the graphene gates. The parameters L_{a}, as well as $\mathrm{P_{in}}(x)$, have been obtained by means of the modal analysis of the structure in Supplementary Figure 1.a, performed using a commercial mode solver, and shown in the Supplementary Note 3.

We used $T_{\mathrm{HC}} = T_{\mathrm{lattice}}$ as boundary condition in $x = \pm L/2$, V = 0 V in $x = -L/2$ and $V = V_{\mathrm{d}}$ in $x = L/2$. The photocurrent is computed as

$$I_{\mathrm{ph}} = \int_0^W J_x\left(x = \frac{L}{2}, z\right) dz \qquad \text{(Supplementary Equation 2)}$$

and the photovoltage V_{ph} is obtained as the drain voltage when I_{ph} is null.

Supplementary Note 2 - Conductivity model

The Seebeck coefficient S and the thermal conductivity k_{HC} have been computed by using the Mott's formula[1,4] ($S = -\frac{\pi^2 k_B^2 T}{3e}\frac{1}{\sigma}\frac{d\sigma}{d\mu_c}$ where k_B is the Boltzmann constant, e the electron charge and μ_C is the chemical potential in the graphene channel) and the Wiedemann-Franz law[1,5] ($k_{HC} = \sigma LT$, L is the Lorentz number). A conductivity model similar to the one proposed in Ref[6] has been used

$$\sigma = \sigma_{\min}\sqrt{1+\frac{n^2}{\Delta n^2}} \qquad \text{(Supplementary Equation 3)}$$

where n is the charge carrier concentration and Δn is the magnitude of the carrier density fluctuations in graphene in the proximity of the charge neutrality point (we experimentally obtain Δn by measuring the residual charge carrier concentration n* at the CNP[7]). By using the relation:

$$n = \frac{1}{\hbar^2 v_F^2 \pi}\mu_C^2 \qquad \text{(Supplementary Equation 4)}$$

($\hbar$ is the reduced Planck's constant, v_F is the Fermi velocity in graphene), we can write:

$$\sigma = \sigma_{\min}\sqrt{1+\frac{\mu_C^4}{\Delta^4}} \qquad \text{(Supplementary Equation 5.a)}$$

$$\Delta = \hbar v_F \sqrt{\pi \Delta n} \qquad \text{(Supplementary Equation 5.b)}$$

We extracted the parameters needed to compute the sheet conductivity curve (Supplementary Equation 5.a) from four-probe and Transfer-Length-Method (TLM) measurements performed in a previous work[8]. A representative curve of the sheet conductance as a function of the gate voltage ($V'_{\text{gate}} = V_{\text{gate}} - V_{\text{CNP}}$, where V_{CNP} is the gate voltage corresponding to the charge neutrality point) is shown in Supplementary Figure 2.a. By using the method proposed by Couto et al.[7] we extracted a residual charge carriers concentration at the CNP $n^* \approx 7\text{x}10^{11}\text{cm}^{-2}$ (Supplementary Figure 2.b) corresponding to a Δ value equal to about 100 meV.

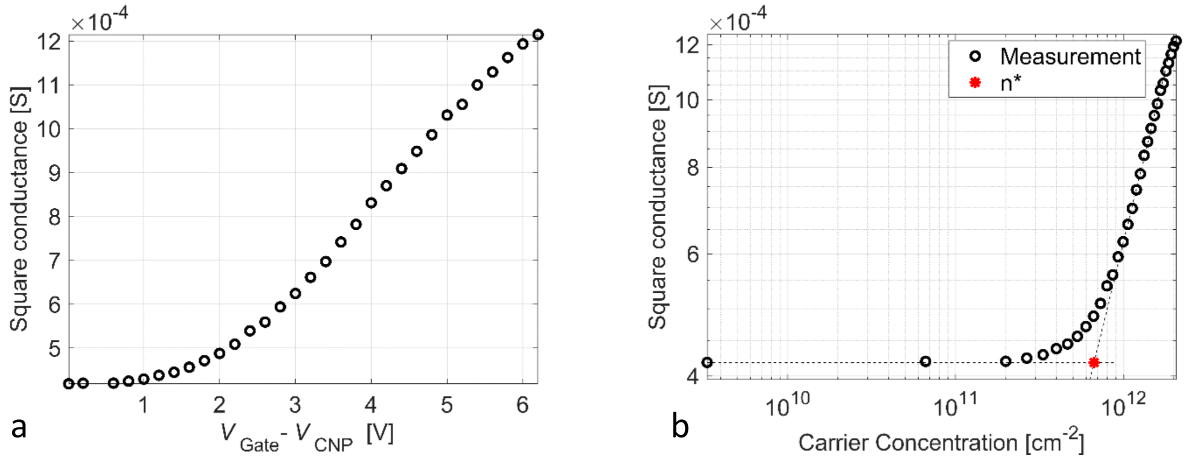

Supplementary Figure 2. Graphene characterization. a. Square conductance of a graphene sample as a function of the gate voltage. Four-probe measurement obtained from a Hall bar structure using 100 nm thick SiN as a gate dielectric for top gating. b. Fitting procedure to extract n*.

The minimum square conductance σ_{min} is close to 5x10^{-4}S (Supplementary Figure 2.a). The contact resistance R_C has been computed from TLM measurements at a chemical potential equal to 180 meV, close to the maximum theoretical responsivity point ($\mu_C = \pm\sqrt[4]{3}\Delta$ = ±130meV). The mean value of R_C is about 500 Ω µm. By using these parameters in Supplementary Equation 5.a, we obtain a sheet resistance equal to 1000Ω/sq. at μ_C=±130 meV. Those values are consistent with the ones of ref[8], where we measured a 1000Ω/sq. sheet resistance at a similar doping level. We assumed the chemical potential to be constant in the regions under the gate electrodes and to smoothly vary in the 150 nm gap region. The photovoltage map in Supplementary Figure 2.d of the main text has been simulated by sweeping the chemical potential on both sides of the junction in a range (-200 meV,200 meV). The corresponding gate voltage has been obtained by using the relation $en \approx C_{\text{gate}}(V_{\text{gate}} - V_{\text{CNP}})$ and $C_{\text{gate}} = \varepsilon_0 \varepsilon_{\text{SiN}}/t_{\text{SiN}}$ is the gate capacitance per unit area (ε_0 is the vacuum permittivity, $\varepsilon_{\text{SiN}} = 6$ is the relative dielectric constant used for SiN and t_{SiN} is gate dielectric thickness).

Supplementary Note 3 - Optical simulation and heat source term

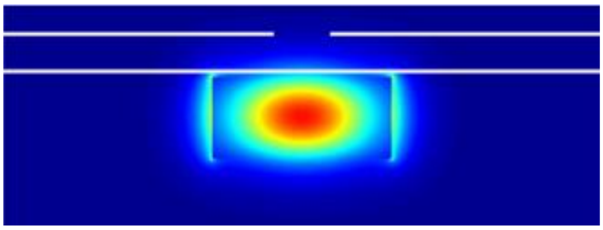

Supplementary Figure 3. Electric field intensity profile of the fundamental TE mode sustained by the waveguide in Fig. S1.a (Si core 480nmx220 nm, λ=1550nm).

The absorption length L_a, the power density $P_{in}(x)$ in the active graphene layer, the parameter η, and the minimum distance of the source-drain contacts from the waveguide to prevent losses and the optimal gate dielectric thickness are obtained from the simulation of the fundamental TE mode (Supplementary Figure 3) of the SOI waveguide with the detector stack (Supplementary Figure 1.a), at a wavelength equal to 1550 nm and propagating in the *z*-direction. The optical properties of the graphene layers have been modeled by using a surface conductivity model[9].

Optical power propagating in an absorbing medium (*z*-direction) undergoes to an exponential attenuation[12]

$$\mathrm{P_{opt}}(z) = \mathrm{P}(z=0)\exp(-\alpha z) = \mathrm{P}(z=0)\exp(-z/L_\mathrm{a}) \qquad \text{(Supplementary Equation 6)}$$

In Supplementary Equation 6 $\alpha = 2\overline{\alpha}$ is the power attenuation constant, $\overline{\alpha}$ is the attenuation constant of the electric field amplitude and $L_a = 1/\alpha$ is the absorption length[10]. The amount of power absorbed between a point in *z* and *z*+*dz* in the active graphene layer, where *dz* is an infinitesimal distance, is, therefore,

$$-\frac{d\mathrm{P_{opt}}}{dz} = \frac{1}{L_\mathrm{a}} P(z=0)\exp(-\frac{z}{L_\mathrm{a}}) \qquad \text{(Supplementary Equation 7)}$$

Since in the case of a waveguide integrated photodetector we have an optical mode, L_a is obtained from the complex propagation constant. Due to the transversal spatial extension of the optical mode, the absorbed power density has a spatial profile along the *x*-coordinate that can be obtained from the *z*-component of the Poynting vector $P_z(x,z=0)$ in the (x,z) plane at $y=0$.

We assume that for an optical mode propagating in absorbing layers of infinite length, the mode optical power P_{in}^* at the detector input is completely absorbed.

$$\int_0^{+\infty}\int_{-\infty}^{+\infty}\left(-\frac{d\mathrm{P_{opt}}}{dz}\right)dxdz = \int_{-\infty}^{+\infty}\frac{1}{L_\mathrm{a}}\mathrm{P_{in}}(x)dx\int_0^{+\infty}\exp\left(-\frac{z}{L_\mathrm{a}}\right)dz = P_\mathrm{in}^* \quad \text{(Supplementary Equation 8)}$$

From Supplementary Equation 8 we obtain the following condition:

$$\int_{-\infty}^{+\infty}\mathrm{P_{in}}(x)dx = \int_{-\infty}^{+\infty}A\frac{1}{2}Re(\mathrm{P_z}(x, y = 0, z = 0))dx = P_\mathrm{in}^* \quad \text{(Supplementary Equation 9)}$$

where A is a normalization constant.

Part of the optical power is absorbed in the graphene gate electrodes and does not contribute to the photovoltage generation. The term η accounts for the fraction of the absorbed power which is effectively used in the photoconversion and is defined as:

$$\eta = \alpha_\text{active layer}/\alpha_\text{total} \quad \text{(Supplementary Equation 10)}$$

We obtained α_total by simulating the detector with the full stack, including gates (Supplementary Figure 1.a and Supplementary Figure 3) and $\alpha_\text{active layer}$ by removing the upper graphene layer. We assumed the presence of the graphene gates does not strongly modify the spatial profile of the optical mode as it may happen with metal gates, and the only parameter that changes in the two simulations is the power absorption.

The heat source term in Supplementary Equation 1.a is, thus, written as:

$$\mathrm{P_{absorbed}}(\mathrm{x,z}) = \eta\frac{\mathrm{P_{in}}(x)\exp\left(-\frac{z}{L_a}\right)}{L_\mathrm{a}} = \eta\frac{P_\mathrm{in}^*}{\int_{-\infty}^{+\infty}\frac{1}{2}Re\left(\mathrm{P}_z(x,y=0,z=0)\right)dx}Re\left(\frac{1}{2}(\mathrm{P_z}\ (x, y = 0, z = 0)\right)\frac{\exp\left(-\frac{z}{L_\mathrm{a}}\right)}{L_\mathrm{a}}$$

(Supplementary Equation 11)

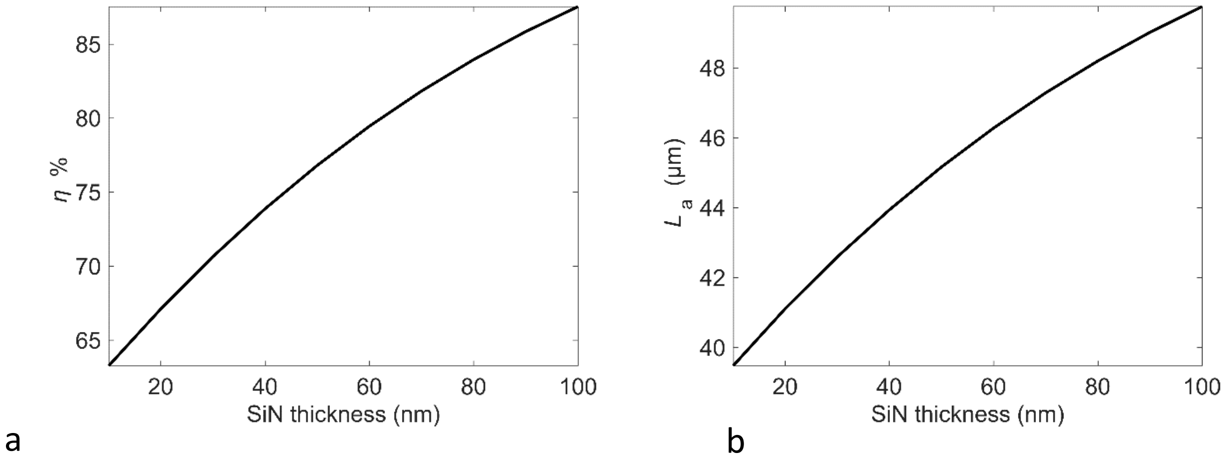

Supplementary Figure 4. η and absorption length a) η% as a function of the SiN gate dielectric thickness. b) Absorption length L_a as a function of the gate dielectric thickness

The absorption length L_a and η depend on the SiN thickness. For a 100nm thick SiN gate dielectric layer almost the 90% of the optical power is absorbed in the active graphene layer and the absorption length is about 50μm (Supplementary Figure 4). We avoided the use of a thicker dielectric layer because, given the low gate capacitance, a very large gate voltage would have been otherwise required to set the maximum responsivity.

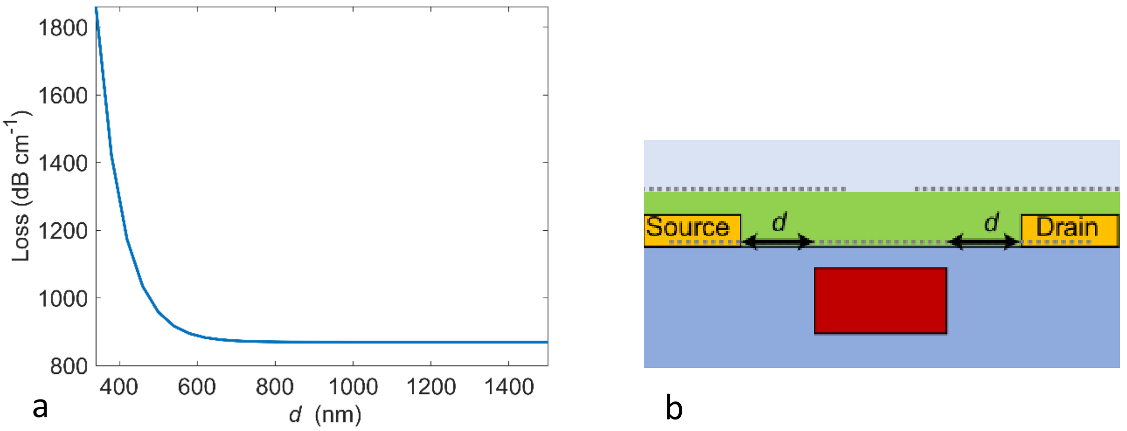

Supplementary Figure 5. Optical loss introduced by contacts. a) Losses of the optical mode as a function of the distance between metal contacts and waveguide. b.) Cross section of the device with contacts.

We simulated the structure also in presence of the source/drain metal electrodes and we varied the distance d (Supplementary Figure 5.b) between the metal contacts and the waveguide. A distance d = 500 nm is sufficient

to prevent extra losses introduced by metals (Supplementary Figure 5.a). Considering the width of the Si waveguide (480 nm) we can use a channel length *L* equal to 1.5µm (see main text).

Supplementary Note 4 - Voltage responsivity drop for device having large width *W*

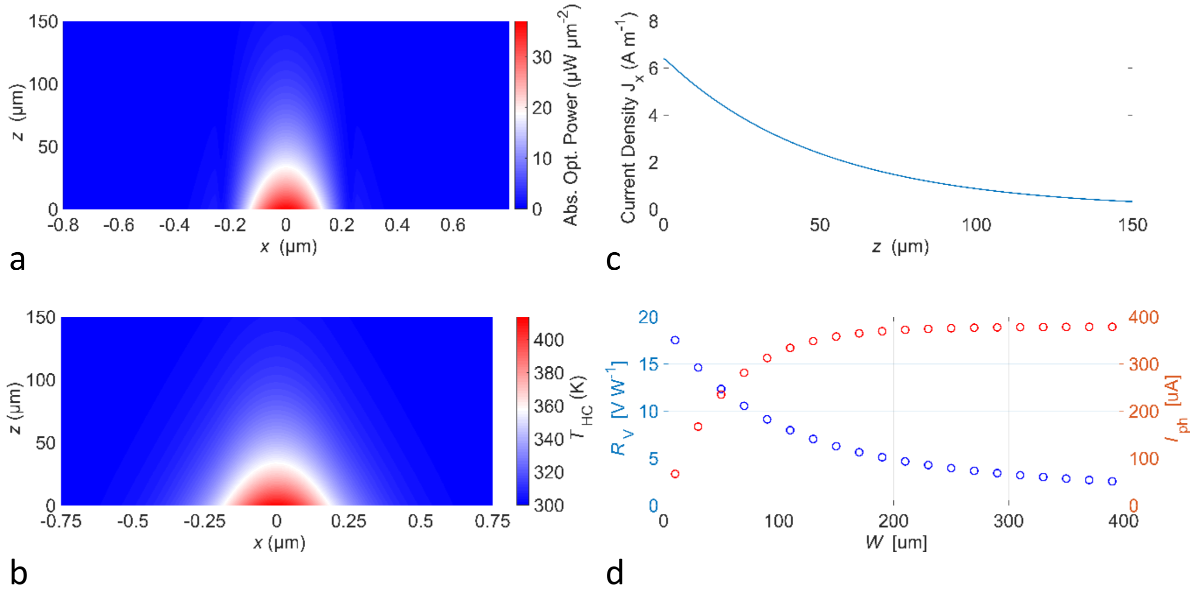

Supplementary Figure 6. Simulation results. a) Heat source as defined in Supplementary Equation 11 for a device having a channel length L=1.5μm, a channel width W=150μm, absorption length L_a≈50μm and P_{in}=1mW. b) Spatial profile of T_{HC} (numerical solution of Supplementary Equation 1) associated to the heat source in Supplementary Figure 5.a. c) x-component of the current density (numerical solution of Supplementary Equation 1) along the drain contact surface (x=L/2), heat source in Supplementary Figure 5.a . d) Photocurrent I_{ph} as defined in Supplementary Equation 2 obtained from the current density in Supplementary Figure 5.c.

The density of absorbed optical power exponentially decreases (exp(-z/La)) along the propagation direction z (Supplementary Equation 11, Supplementary Figure 6.a). The HCs temperature undergoes to the same exponential damping (Supplementary Figure 6.b) as the current density (Supplementary Figure 6.c). For z>>La (La≈50μm in Supplementary Figure 6) the photocurrent density Jx weakly contributes to the total photocurrent $I_{\text{ph}} = \int_0^W J_x\left(x = \frac{L}{2}, z\right) dz$. The photovoltage Vph and the voltage responsivity RV=Vph/Pin decrease with the channel width W (Supplementary Figure 6.d, red curve). Indeed, Vph (the open circuit voltage of the current-voltage characteristic under illumination) and I_{ph} (the short circuit current) are linked through the relation $V_{ph}=RI_{ph}$[11]. For large channel width (W>>L_a) the photocurrent saturates (Supplementary Figure 6.d, blue curve) and the device resistance is reduced, therefore, the voltage responsivity drops (Supplementary Figure 6.d, red curve).

Supplementary Note 5 - Series Impedance of the photodetector

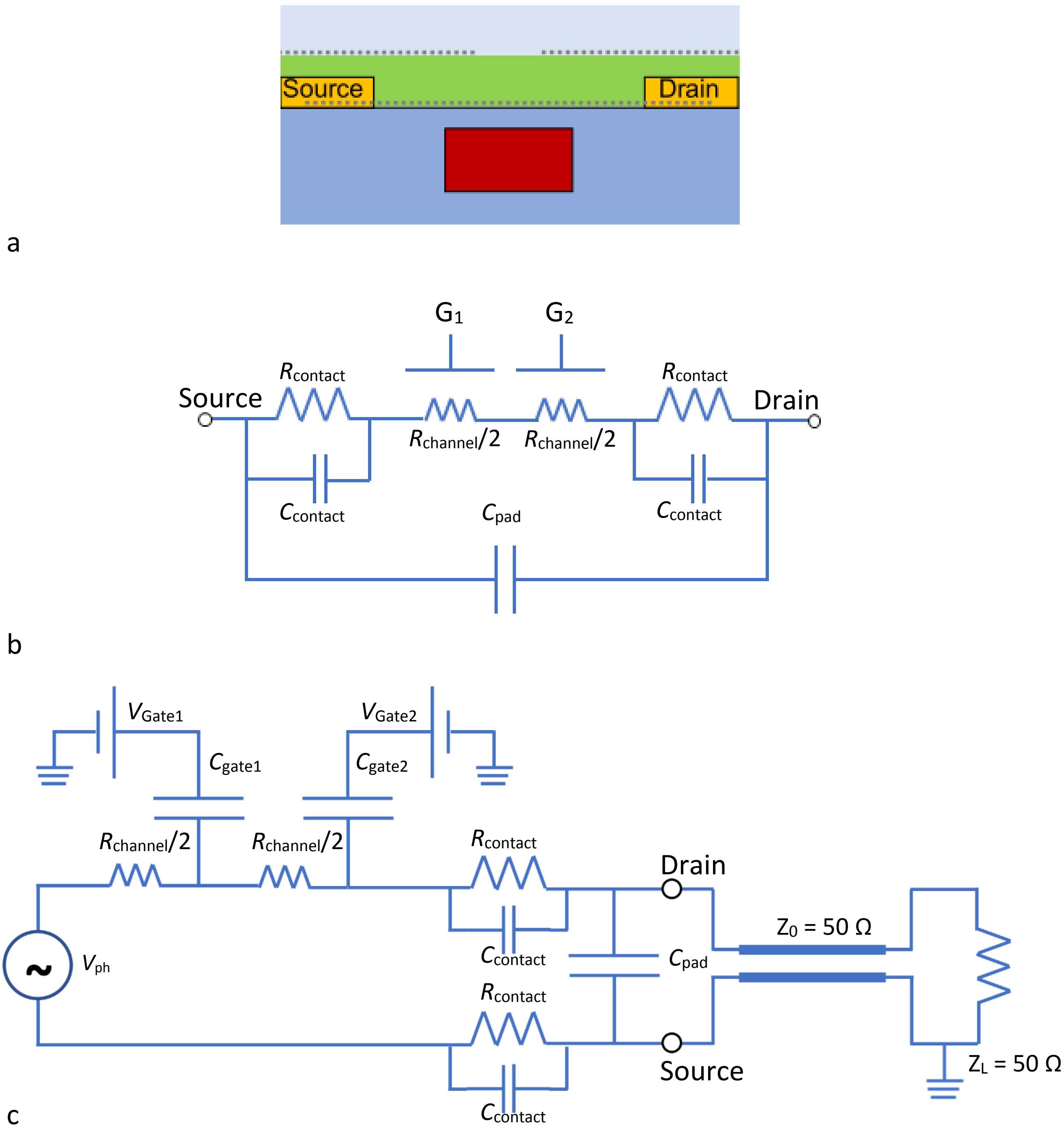

Supplementary Figure 7. Equivalent electrical model. a) Cross section of the device and b) schematic representation of the resistance and capacitance distribution in the graphene channel under the gate electrodes. c) Equivalent electric circuit of the PTE photodetector when connected to a 50 Ω load through a lossless transmission line (characteristic impedance $Z_0 = 50\ \Omega$).

Supplementary Figure 7c shows an equivalent electrical circuit model of the graphene photodetector. We modelled the photodetector as a voltage source with a series impedance. The capacitances due to the presence of the gates may affect the frequency response of the photodetector and introduce a cut-off frequency reducing the optoelectronic bandwidth. The electrical modeling is complicated by the fact that the lowest gate capacitor plate is a portion of the graphene active channel (Supplementary Figure 7.a-b). We model the gate capacitive effect as two capacitors placed in the half and at the end of the graphene channel (Supplementary Figure 7.c).

$$R_{\mathrm{contact}} = \frac{R_{\mathrm{c}}}{W} \qquad \text{(Supplementary Equation 12.a)}$$

$$R_{\mathrm{channel}} = \frac{R_{\mathrm{ch}} L}{2W} \qquad \text{(Supplementary Equation 12.b)}$$

$$C_{\mathrm{gate,1,2}} = \frac{\varepsilon_0 \varepsilon_{\mathrm{SiN}}}{t_{\mathrm{SiN}}} \frac{L}{2} W \qquad \text{(Supplementary Equation 12.c)}$$

where R_c=500 Ω µm and R_{ch} = 1000 Ω/sq.[8], $LW/2$ is the area of a single gate capacitor plate. The other parameters like the contact capacitance C_C and the pad capacitance C_{pad} are assumed to be similar to the ones reported in ref[12].

$R_{channel}$	$R_{contact}$	$C_{gate,1,2}$	$C_{contact}$	C_{pad}
30 Ω	10 Ω	20 fF	0.12 pF	1.2 fF

Supplementary Table 1. Electrical parameters of the circuit in Supplementary Figure 6.c computed for a device geometry L = 1.5µm and W = 50 µm or taken from ref.[14]

We simulated the frequency response of the circuit in Supplementary Figure 7.c. (Supplementary Figure 8) by using the parameters in Table I. The frequency response is almost flat for f < 100 GHz (attenuation of 0.7 dB at f = 100 GHz with respect to the low frequency value). In this frequency range the device behavior is mainly determined by the resistive component of its impedance which limits the power transfer to the 50 Ω load. Since our experimental set-up is limited to f < 100 GHz, we optimized the PTE photodetector by considering only the channel and the contact resistance and neglecting the capacitive effects (simplified circuit in Supplementary Figure 9).

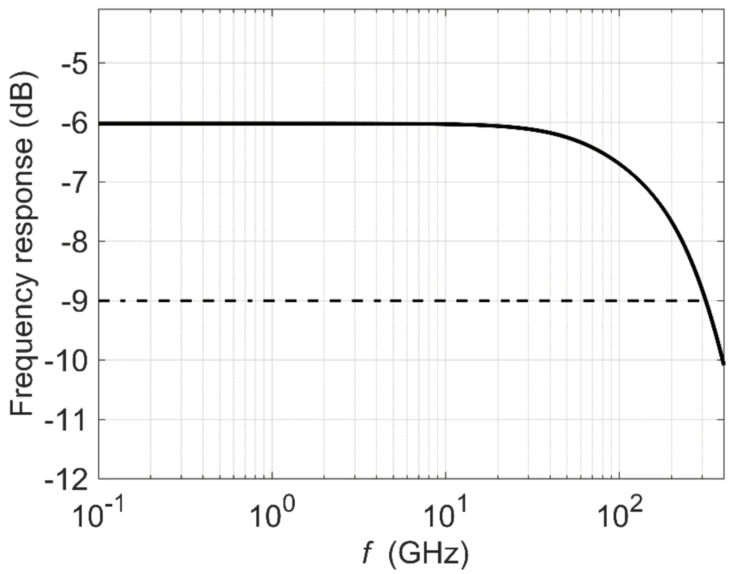

Supplementary Figure 8. Frequency response (output on Z_L) of the circuit in Supplementary Figure 6c using the parameters in Supplementary Table 1.

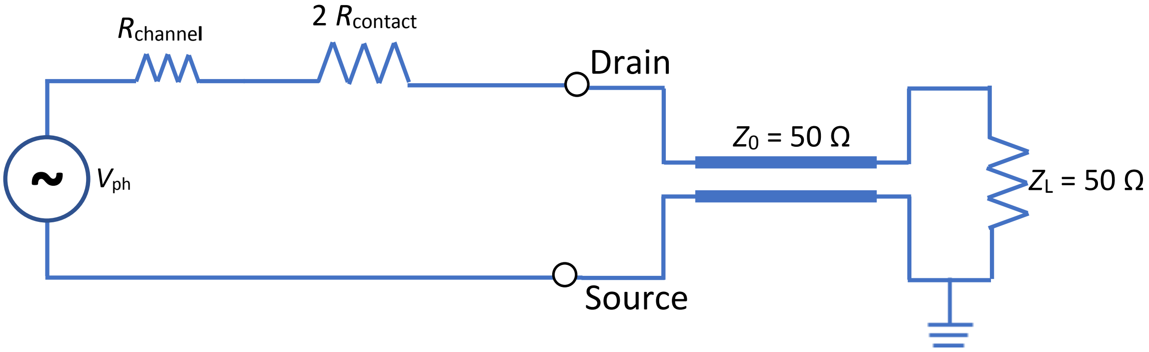

Supplementary Figure 9. Simplified equivalent circuit used in the main text for the series impedance optimization

SUPPLEMENTARY REFERENCES

1. Song, J. C. W. W., Rudner, M. S., Marcus, C. M. & Levitov, L. S. Hot carrier transport and photocurrent response in graphene. *Nano Lett.* **11**, 4688–4692 (2011).

2. Thomson, W. 4. On a Mechanical Theory of Thermo-Electric Currents. *Proc. R. Soc. Edinburgh* **3**, 91–98 (1857).

3. Woessner, A. *et al.* Near-field photocurrent nanoscopy on bare and encapsulated graphene. *Nat. Commun.* **7**, 1–7 (2016).

4. Cutler, M. & Mott, N. F. Observation of anderson localization in an electron gas. *Phys. Rev.* **181**, 1336–1340 (1969).

5. Franz, R. & Wiedemann, G. Ueber die Wärme- Leitungsfähigkeit der Metalle. *Ann. Phys.* **165**, 497–531 (1853).

6. Shiue, R. J. *et al.* High-Responsivity Graphene-Boron Nitride Photodetector and Autocorrelator in a Silicon Photonic Integrated Circuit. *Nano Lett.* **15**, 7288–7293 (2015).

7. Kim, S. *et al.* Realization of a high mobility dual-gated graphene field-effect transistor with Al2O3 dielectric. *Appl. Phys. Lett.* **94**, 062107 (2009).

8. Giambra, M. A. *et al.* High-speed double layer graphene electro-absorption modulator on SOI waveguide. *Opt. Express* **27**, 20145 (2019).

9. Couto, N. J. G. *et al.* Random Strain Fluctuations as Dominant Disorder Source for High-Quality On-Substrate Graphene Devices. *Phys. Rev. X* **4**, 041019 (2014).

10. Zhong, H., Zhang, Z., Xu, H., Qiu, C. & Peng, L. M. Comparison of mobility extraction methods based on field-effect measurements for graphene. *AIP Adv.* **5**, (2015).

11. Hanson, G. W. Dyadic Green’s functions and guided surface waves for a surface conductivity model of graphene. *J. Appl. Phys.* **103**, 064302 (2008).

12. Ghione, G. *Semiconductor Devices for High-Speed Optoelectronics*. (Cambridge University Press,

2009). doi:10.1017/CBO9780511635595

13. Gabor, N. M. *et al.* Hot carrier-assisted intrinsic photoresponse in graphene. *Science (80-.).* **334**, 648–652 (2011).

14. Awan, S. A. *et al.* Transport conductivity of graphene at RF and microwave frequencies. *2D Mater.* **3**, 0 (2016).
